# The Excito-Secretory System

**Published:** 1857-10

**Authors:** J. Adams Allen

**Affiliations:** Formerly Professor of Therap., Mat. Med. and Juris. in the Indiana Medical College; and late Professor of Physiology and Pathology, and acting Prof. of Mat. Med. in the University of Michigan


*The Excito-Secretory System.—Dr. H. F. Campbell—Marshall Hall—Alleged Priority of Discovery.* By J. ADAMS ALLEN, A M., M. D., Formerly Professor of Therap., Mat. Med. and Juris. in the Indiana Medical College; and late Professor of Physiology and Pathology, and acting Prof. of Mat. Med. in the University of Michigan.

It appears from a late number of the London Lancet that M. Hall recognizes to a certain extent the priority of Dr. Campbell. His words are these:

" I arrive at this conclusion : the idea and the *designation* of an excito *secretory* action belongs to Dr. Campbell, but his details are limited to pathology and observation. The elaborate experimental demonstration of reflex excito-secretory action is the result of the experimental labors of M. Claude Bernard.

" My own claim is of a very different character, and I renounce every other. It consists in the vast *generalization* of excito-secretory action throughout the system.

" I trust Dr. Campbell will be satisfied with my adjudication. There is in the excito-secretory function, as applied to pathology, an ample field of inquiry for his life's career, and it is indisputably—HIS OWN. He first detected it, gave its designation and saw its vast importance."

M. Hall thus far, freely and fully admits the priority of Dr. Campbell, and the latter gentleman bases his claim upon the date, May, 1850.

I shall undertake to show that this same doctrine was FIRST publicly announced and illustrated in my lectures at the Indiana Medical College in November 1848, and thenceforth continuously during the continuance of my public teaching before the several classes of that College, and also the medical classes of the University of Michigan until my connection with that institution "expired by limitation" in 1854. My own manuscript, containing this doctrine, was written in May or June 1848.

In proof of this proposition I shall adduce the ancient manuscript which I have fortunately the means of proving as to date, in the most satisfactory manner; and, secondly, the evidence of students then in attendance upon my lectures, and now some of them occupying high professional positions.

As the lapse of years has scattered the various classes throughout different sections of the country, and the addresses of but few are now known to me, I would crave permission to take this mode of inviting all graduates or students of the Indiana Medical College during the session of 1848-9, and 1849-50 to forward me their present P. O. address, that my claims may be fully substantiated by their evidence.

Of this one thing I am fully convinced, that the enunciation of the Excito-Secretory principle as *new*, will fall upon the ears of such students as have from time to time listened to my lectures, with something like astonishment.

The manuscript itself was never more than a text to extended explanatory remark upon the subject.

I do not claim the nomenclature " Excito-*Secretory*" as invented by me, and shall take occasion in the proper place to remonstrate against the limitation of the great phenomena involved, by this merely arbitrary term. Analogically it is convenient in contradistinction to the term excito-motory, but mere analogy too often leads us into error to be a safe guide, even in nomenclature.

What I do claim—*is the great generalization, that the excito influence is followed by a reflex change in which the effect is* NOT A MOTION *but* A MODIFICATION OF VASCULAR AND UTRIENT ACTION. *That this effect takes place* BY MEANS OF THE DOUBLE NERVOUS ARC. A vast number of therapeutic phenomena are thus explained.

As M. Hall is kind enough to allow Dr. Campbell the credit of having discovered the "excito-secretory function, as applied to pathology," perhaps he may be led to recognize my own claim to the discovery of this principle as applied to *therapeutics.* I shall not be content with this.

In my course upon "General Therapeutics" the subject of "Counter-Irritation" came under review.

After considering the numerous cases wherein nature seems to set up this process in the prevention of disease—or at least where when the external disease subsides, internal derangement quickly supervenes; which, again, is relieved by re-establishment of the external difficulty, (e. g. ulcers upon the legs, *fistula in ano*, otorrhœa, cutaneous eruptions, &c., &c.) I passed to consider the issue, seton, moxa, epispastic, &c.

After giving several hypotheses from various sources, of their action therapeutically, and particularly Eberle's view, the lecture proceeds as follows:

"This view," (Eberle's) "of the subject after all, that it is the most plausible one which has been given, is fraught with some difficulties. Why should a blister upon the thorax in pneumonia be more beneficial than one upon the arm? Or why should an epispastic over the abdomen in enteritis be preferable to one upon the thigh? The parts although nearer together topographically, are anatomically much farther apart. Hence, reasoning, anatomically, or from the distribution of the blood vessels, we should be inclined to reverse our application."

"But experience, in this instance, as in many others contravenes analogy, and to its mandates we must succumb. How is the anomaly to be explained?"

"Certainly not by Hunter's 'contiguous sympathy' for this explains nothing: there is no short cut passage between the outer vesicated surface, and the inner inflamed organ—neither by anastomosing vessels nor nerves."

"* Hence the impression MUST BE *transmitted to the nervous centres*,

*Here is written upon the margin of the manuscript, "*Original Explanation.*"

*and thence reflected to the affected organ; in other words, the influence is primarily exerted upon the cerebro-spinal system, and secondarily upon the internal affected organ.*"

This is the gist of the whole matter, and the point consists in the recognition of reflex cerebro-spinal action which in the instances adduced give rise to a *molecular* or *integral* change in the inflamed tissue—and not a muscular contraction.

In the oral elaboration of this principle was suggested an idea which does not even now appear to have occurred to either M. Hall or Dr. Campbell, viz: *The motor effect is merely* SECONDARY, *and not a necessary part of the action of the nervous arc.*

The molecular or integral composition or decomposition at the distal extremity of the reflex nerve is the real element—the motion, secretion or change of vascular or nutrient action, production or abatement of inflammation or other change of the organ supplied by the nerve (reflex), being altogether *secondary or incidental.*

THE EFFECT IS MOTORY, IF CONTRACTILE FIBRE BE PRESENT!

THE EFFECT IS SECRETORY, IF SECRETING ORGANS BE SUPPLIED!

THE EFFECT IS SENSATION, IF SENSITIVE NEURINE BE REACHED!

THE EFFECT IS PERCEPTION, OR INFELLECTION, IF THE ORGAN THEREOF BE IN CONNECTION WITH THE REFLEX NERVE!

I have said that the term excito-secretory is objectionable; the term excito-motory is equally so. The terms "*external* and *internal* distaltic action," are equally so. Whether internal or external *the modus is the same.*

Heat will melt iron, explode gunpowder, harden clay and destroy or fix colors.

Are these several results indicative of any *diversity* in the force of heat itself? Are they not rather indicative merely of *variety in the bodies acted upon*?

So is it with the excitor force which commands the tiny fibrillæ and cells of the nervous arc. They receive and transmit but the *single impulse*—and in a *single* manner.

*The effect produced, then depends upon the* STRUCTURE AND CONDITION *of the organ reached.*

The single tubule or fibre of nerve may whilst conducting its peculiar impulse touch, here upon a muscular fibre and call its contractile elements into energetic action, and there upon a cell or group of cells and their growth, maturation and dissolution are hurried on; and so on through the category of vital changes.

But again, this influence *is* NOT *confined to the mere increase of action*, as the term EXCITOR might perhaps suggest.

The reverse may take place—the excitor may rather become the *depressor.*

It would be as correct to say the depressor-motory, the depressor-secretory, as to say the excitor-*idem.*

Physiology, Pathology and Therapeutics are full of illustrations of this fact.

The excito-secretory function is not in fact distinct from the excito-motory—neither is the depresso-secretory from the depresso-motory.

We might with equal propriety speak of a modification in essence of the electro-galvanic circuit when at one time, by ingenious mechanism, it is caused to print a telegram, and at another to decompose a mineral—at another to kindle the Drummond light.

The 22d of December, 1845, the Surgical Napoleon of America uttered these words in a lecture which now (as they did then), strike me as prophetic: "We have got to the end of the *vascular* system—and just beginning the *nervous* system."

It is useless to lumber up our already Babel-nomenclature with new and inexpressive barbarisms.

The terms excitor and depressor do not include the functions of the first arm of the nervous arc. Pathologists and therapeutists have been obliged to recognize something more than the mere increase or diminution of action.

*Qualitative* changes are fully as important, if not more so, than simple *quantitative* ones—and these cannot be properly designated by the terms proposed by Messrs. Hall and Campbell.

There is the recipient segment, the impressible centre, and the reflex conductor, in all the known machinery of the nervous tissue. The resultant effect depends upon the nature of the structure acted upon.

In this view how important is seen to be the material constitution of the parts acted upon.

The *vis nervosa*, or highest relation of force with which we are acquainted, has not to do with intangible things, but requires for its appropriate action the presence of particular material structures, of particular chemical compounds or elements, and a certain arrangement.

A clear comprehension of the phenomena of nervous action demonstrates the necessity of something more than shadowy remedies for existing derangements. But of this hereafter—that the limits of the present paper be not too greatly extended.

One thought more in connection with the views of M. Hall.

"There is" says he, "perhaps, not a point in the general cutaneous surface in which tetanus—an excito-*motor* effect—may not originate; there is scarcely a point in which internal inflammation—an excito-secretory effect—may not be excited."

Turn now to any late treatise upon the physiology of muscular contraction, and we find the doctrine of the inherent "irritability" of the muscular fibre—the nervous system merely calls this into action—as it does any stimulus tending to disturb its integral structure.

The reflected impulse disturbs the molecular structure and contraction results as it would from the application of a chemical agent or mechanical appliance. If continuous, inflammation or even destruction of the tissue may ensue.

Where is tne line of demarcation between the two effects; or, rather, is there any such line or real distinction? Correctly speaking all nutrition is but secretion—all secretion is but nutrition. Contraction (and therefore motion) is but an incident in the nutrition or molecular change of muscle.

The nutrition of the liver or of any other glandular organ is precisely identical in its ultimate elements.

The nervous act is in each instance the same.

Where then is the assumed distinction?

It is perhaps safe to assume as probable that parts supplied with cerebro-spinal nerves, exhibit more definiteness in their reflex phenomena than those supplied mainly by fibres from the ganglionic chain. *But it remains to be proved that there is any more real difference between these two classes of nerve fibres, than exists between striated and non-striated muscular fibres.*

For the present I leave the subject by soliciting attention to the real point at issue, which is: that the afferent nerve, the central organ, and the efferent nerve constitute the true system: that the terms "excito-secretory and excito-motory systems" are ill-chosen and erroneous as respectively too limited in signification and tending to misapprehension of the real simplicity of the true system which I first publicly pointed out in 1848.

---

LETTERS IN EVIDENCE OF THE NATURE AND DATE OF MY PUBLIC TEACHING.

(From Robert C. Kedizie, A. M., M. D.)

VERMONTVILLE, Mich. July 29th, 1857.

Dr. J. A. ALLEN.

DEAR SIR:—Your favor of the 23d was received yesterday, and I reply by first mail.

In reply to your inquiry, I would state that I remember distinctly the matter to which you allude. Your reasoning upon the therapeutic action of a blister in pneumonia (also of a blister to the abdomen in enteritis), I remember as distinctly as though I listened to you but yesterday. Your accounting for it by the double nervous arc—analogous to the excito-motory arc, I remember with equal distinctness; though I think you did not name it then the excito-secretory system. * * * I also remember your rejection of the proposition of "contiguous sympathy."

I could identify your manuscript lectures in any court of justice in the land. It would be impossible for them to be essentially altered and I not detect it.

I should have noticed the injustice of Dr. Campbell's claim to priority, but the fact is &c., &c. (Here follow certain personal explanations unnecessary to the present purpose. A.)

Ever Yours Truly, R. C. KEDZIE.

[Dr. Kedzie was a member of the class of 1849 at Laporte, Ia., Medical College—afterwards a graduate of, and Demonstrator of Anatomy in the University of Michigan. Dr. K. had constant access to my manuscripts and took copious notes.]

---

(From Joseph B. Hull, M. D.,)

Lansing, Mich., July 28th, 1857.

Dear Sir:—I received yours of the 24th yesterday, and am glad of an opportunity of referring to the instructions received whilst I listened to your lectures both at Laporte (Ia. Med. Coll.) in '49 and Ann Arbor in '50. I shall not be under the necessity of referring to notes to refresh my memory upon most subjects treated of by you.

In regard to the subject of controversy between Messrs. Hall and Campbell about priority in establishing the doctrine ef excito-secretory nervous action—if they cannot date back of 1850, they certainly cannot claim its introduction to the consideration of the profession, as you in your lectures at Laporte in 1849 avowed the principle in treating upon the subject of counter-irritation.

You then rejected the theory of "sympathy of contiguity" and attributed the effects of blisters upon the chest and abdomen in pneumonia and enteritis (as well as near other local inflammations) to the production of an internal change in the vascular and nutrient action similar to the excitor and reflex action of the nervous system, and I think you explained the phenomenon by reference to the double nervons arc. I hope you will succeed in placing the priority of broaching the above principle where it belongs.

Respectfully Yours, J. B. HULL.

[Dr. Hull can also identify the manuscript.]

---

(From Charles P. Marsh, M. D.)

Holland, Mich., Aug. 2d, 1857.

Dear Dr.:—In reply to yours of the 23d ult.

I can identify your manuscript lectures as written in the spring and summer of 1848, and first delivered at the session of the Indiana Med. College 1848–9.

I remember distinctly your explanation of the action of counter-irritants by means of the double nervous arc. You rejected the theory of "sympathy of contiguity" and claimed as an original view the principle that their action was analogous to the excito-motory of M. Hall, but that the reflex effect was not a motion but a modification of the nutrition of the internal organ.

You gave many illustrations of this mode of action throughout the entire course.

The same doctrine was also taught by you in the University of Michigan, whilst I attended the sessions of '50 and '51, '51 and '52, both in connection with your lectures upon Therapeutics and Physiology. * * *

Yours Cordially, CHARLES P. MARSH.

[*Medical Independent.*]

